# Use and Acceptance of Electronic Communication by Patients With Multiple Sclerosis: A Multicenter Questionnaire Study

**DOI:** 10.2196/jmir.2133

**Published:** 2012-10-15

**Authors:** Rocco Haase, Thorsten Schultheiss, Raimar Kempcke, Katja Thomas, Tjalf Ziemssen

**Affiliations:** ^1^Multiple Sclerosis Centre DresdenDepartment of NeurologyUniversity of Technology DresdenDresdenGermany

**Keywords:** Multiple sclerosis, computers, email, mobile phone, health information seeking, disease management, communications media, health information technology

## Abstract

**Background:**

The number of multiple sclerosis (MS) information websites, online communities, and Web-based health education programs has been increasing. However, MS patients’ willingness to use new ways of communication, such as websites, mobile phone application, short message service, or email with their physician, remains unknown.

**Objectives:**

We designed a questionnaire to evaluate the a priori use of electronic communication methods by MS patients and to assess their acceptance of such tools for communication with their health care providers.

**Methods:**

We received complete data from 586 MS patients aged between 17 and 73 years. Respondents were surveyed in outpatient clinics across Germany using a novel paper-and-pencil questionnaire. In addition to demographics, the survey items queried frequency of use of, familiarity with, and comfort with using computers, websites, email, and mobile phones.

**Results:**

About 90% of all MS patients used a personal computer (534/586) and the Internet (527/586) at least once a week, 87.0% (510/586) communicated by email, and 85.6% (488/570) communicated by mobile phone. When asked about their comfort with using electronic communication methods for communication with health care providers, 20.5% (120/586) accepted communication by mobile Internet application or short message service via mobile phone, 41.0% (240/586) by websites, 54.3% (318/586) by email service, and 67.8% (397/586) by at least one type of electronic communication. The level of a priori use was the best predictor for the acceptance of electronic communication with health care providers. Patients who reported already searching online for health information (odds ratio 2.4, *P *< .001) and who had already communicated with a physician through a website (odds ratio 3.3, *P *= .03) reported higher acceptance for Web-based communication. Patients who already scheduled appointments with their mobile phones (odds ratio 2.1, *P *= .002) were more likely to accept the use of mobile phone applications or short message service for communicating with their physician.

**Conclusions:**

The majority of MS patients seen at specialist centers already use modern communication technology regularly. New forms of electronic communication appear to have high levels of acceptance for exchanging information about MS between patients and health care providers. Such methods should be integrated into eHealth services such as electronic health records and patient relationship management systems.

## Introduction

The way people retrieve health information has changed due to an enormous proliferation of new media technologies and a tremendous growth in health information being available online [1-3]. The Pew Internet & American Life Project subsequently found that 56% to 64% of Americans used online sources of health information [4]. In 2003, 23% of Germans searched online for health-related topics sometimes or often [5]. This trend may be increasing, as studies reported an increase from 44% to 57% in use of the Internet for health purposes in Germany between 2005 and 2007, with the highest use among young women and city dwellers [3,6-9]. Due to the high incidence of multiple sclerosis (MS) in young women (about 70%) showing their first symptoms between the ages of 20 and 40 years, MS patients may be early adopters of emerging eHealth trends [10-13]. To date, research has primarily focused on what the Internet offers to MS patients and how existing health information resources are being accessed. However, studies examining the acceptability of new media such as websites or mobile phone applications for communication with health care providers by MS patients are lacking.

Two studies investigating online information sources about MS in English and German reported variable quality [14,15]. In addition to accessing traditional static information websites, patients can also generate and share their health information with peers and participate in electronic health records for the self-management of disease, with mainly positive results [4,16-21]. Such online communities can also support research, such as the development of a self-report questionnaire to quantify MS patients’ adherence to treatment [22]. Other studies reported the benefits of testing cognitive functions of MS patients online, including better availability and accessibility than with traditional methods [23-25].

Research suggests that MS patients retrieve information about their disease and their physicians online before and after their medical visit, especially before the initial consultation due to potentially high information needs in the early course of the disease [11,26,27]. This may influence patients’ adherence to treatment and their coping styles, and may lead to greater patient empowerment [3,11,28-31], thereby altering the physician-patient relationship [28,32,33]. The observed benefits of new media may have raised the acceptance of eHealth in medicine, but concerns remain such as the digital divide (inequality of access to new communication technologies) and concerns about the security and confidentiality of sharing health data online.

To determine the needs of and future options for patient education and patient–physician communication via new media, we sought to examine the current use of new types of communication (eg, mobile phone applications) and willingness to use these for medical management among MS patients. We hypothesized that patients who were already familiar with electronic communication devices would be more likely to adopt them for communication with health care providers and that the majority of MS patients would already be using an array of electronic communication devices.

## Methods

### Participants

The present study was a multicenter survey conducted in the Multiple Sclerosis Center Dresden (Dresden, Germany), the Multiple Sclerosis Center Stuttgart (Stuttgart, Germany), and several neurological outpatient centers of NeuroTransData GmbH (Neuburg, Germany) between 2009 and 2010. Patients with clinically definite MS according to the McDonald criteria were asked to attend the survey in each participating center over a period of 2 weeks [34]. We targeted recruitment for a minimum of 500 fully completed questionnaires. Overall, 591 of 650 (90.9%) ambulatory MS patients who were asked to participate enrolled in the study. They were surveyed by the final version of the paper-based, self-developed questionnaire during outpatient clinic visits. Prior to study entry, each patient provided a written informed consent and was free to withdraw from the survey at any time for any reason without consequences on the care provided. Because this study involved minimal risk and no personally identifiable information, ethics committee approval was not required.

### Questionnaire

The 18 items in the questionnaire were developed by a scientific advisory board consisting of physicians, psychologists, and computer scientists. The questionnaire surveyed the frequency and nature of personal computer, website, email, and mobile phone use by MS patients, as well as demographic characteristics. Specifically, the questionnaire included 4 demographic items about age, sex, time since MS onset (in years), and residential area (by postal code) (items 1–4), 5 items about computer use (items 5–9), 5 items about Internet use (items 10–14), and 3 items about mobile phone use (items 15–17). We added 1 item about the respondent’s general attitude toward using new media for communication with health care providers (item 18) (see Multimedia Appendix 1).

### Statistical Analysis

Patients were assigned to the *rural area *group (population of place of residence <100,000) or to the *city *group (population of place of residence ≥100,000) based on postal code (questionnaire item 3) [35,36]. We determined disease duration (in years) by calculating the difference between MS diagnosis (questionnaire item 4) and the year of study entry. Use of each medium at least once a week indicated a sufficient frequency for future communication with health care providers. New media acceptance was reported for patients accepting at least one of three types of new media (website, email, and mobile Internet application or short message service) for communication with health care providers. To analyze the influence of age on the a priori use of each medium, we grouped respondents by a median split (younger half: 17-40 years, n = 283; older half: 41-73 years, n = 298).

All statistical comparisons were 2-tailed, and a *P *value of <.05 indicated statistical significance. We used IBM SPSS version 19.0 (IBM Corporation, Somers, NY, USA) for all statistical computations. Acceptance was evaluated by a logistic regression model for each new media type (website, email, mobile Internet application or short message service) using demographic variables and items of specific media type and reporting goodness of fit (Hosmer-Lemeshow test; chi-square) and effect size (odds ratio). Differences in categorical variables were assessed with a chi-square test for between-participants effects and with McNemar test for matched pairs.

## Results

### Characteristics of the Survey Population

In the statistical analysis, we included 586 of 591 MS patients who submitted answers to the last survey question (questionnaire item 18). The majority of study patients were female (408/586, 69.6%) with a mean age of 40.93 (SD 10.84) years (5 missing answers), comparing well with typical population statistics of patients with MD [37]. A total of 6 in 10 patients (336/586, 57.3%) lived in a city area with a population of 100,000 people or more. The disease duration averaged 8.60 (SD 6.52) years since onset (7 missing answers). In all analyses, we observed no impact of residential area (rural area, town, or city) on any item response. Furthermore, patients did not differ between states of the former German Democratic Republic (Saxony) and the Federal Republic of Germany (Baden-Württemberg).

### Computer Use

The vast majority of MS patients (558/586, 95.2%) had access to a computer and 75.4% (442/586) of them personally owned one (Table 1); 70.8% (415/586) used it daily and 91.1% (534/586) used it at least weekly. Men reported higher rates of general use (*P *< .001), computer ownership (men: 151/178, 84.8%; women: 291/408, 71.3%; *P *< .001), and experience in the installation of new software (men: 144/178, 80.9%; women: 231/408, 56.6%; *P *< .001). Nevertheless, computer use was widespread among women. Most patients (509/586, 86.9%) reported acquainting themselves quickly with new software. Younger MS patients tended toward a greater (*P *< .001) and more diversified use of computers (emailing: *P *= .003; browsing the Internet: *P *= .007; chatting: *P *< .001; having already installed new computer programs: *P *< .001; and becoming quickly acquainted with new computer programs: *P *< .001). In all subgroups of MS patients, about 40% (62/178, 34.8%, to 170/408, 41.7%) reported using a computer to retrieve information about their disease. All results are summarized in Multimedia Appendix 2.

**Table 1:** Computer use by patients with multiple sclerosis (MS) (n = 586).

Computer use		%	n
**Frequency of computer use**			
	Several times a day	46.6	273
	Daily	24.2	142
	Several times a week	15.0	88
	Once a week	5.3	31
	Rarely or never	8.9	52
**Computer ownership or shared access**		95.2	558	
	Own a computer	75.4	442
**Type of regular computer use**			
	Browsing the Internet	81.9	480
	Emailing	81.7	479
	Word processing	62.6	367
	Getting information about MS	39.6	232
	Chatting	12.5	73
**Quick familiarization with a new computer program**			
	Definitely applies to me	29.0	170
	Mostly applies to me	33.6	197
	Slightly applies to me	24.2	142
	Does not apply to me	13.1	77
	Installation of computer programs	64.4	375

### Internet Use

About 94% of MS patients (551/586) reported they had Internet access (Table 2). Men browsed websites more frequently (*P *= .009), whereas the number of male and female nonusers was similar. The pattern was the same in the case of reading emails (*P *= .002) and sending emails (*P *= .03). Young MS patients more frequently than older patients performed tasks such as browsing (*P *< .001), chatting (*P *< .001), and emailing (reading: *P *= .02; sending: *P *= .004). MS-related information seeking was reported by between 35.2% (105/298) and 38.9% (110/283) among all subgroups of MS patients. A small number of patients had already communicated with their physician (22/586, 3.8%) or with other patients (29/586, 5.0%) via the Internet (Table 2).

**Table 2:** Internet use by patients with multiple sclerosis (MS) (n = 586).

Internet use		%	n
**Frequency of browsing websites on the Internet**			
	Several times a day	34.6	203
	Once a day	25.9	152
	Several times a week	23.2	136
	Once a week	6.1	36
	Rarely or never	10.1	59
**Internet access at home**			
	Broadband access	69.6	408
	Low-speed access	20.1	118
	No access	6.0	35
	Access type unknown	4.3	25
**Type of regular Internet use**			
	Browsing websites	81.4	477
	Getting information about MS	37.2	218
	Video chatting	11.1	65
	Chatting	10.2	60
	Communicating with other MS patients	5.0	29
	Communicating with physician	3.8	22
**Frequency of sending emails**			
	Several times a day	31.1	182
	Once a day	15.4	90
	Several times a week	23.0	135
	Once a week	11.6	68
	Rarely or never	18.9	111
**Frequency of reading emails**			
	Several times a day	37.2	218
	Once a day	23.2	136
	Several times a week	17.4	102
	Once a week	9.2	54
	Rarely or never	13.0	76

### Mobile Phone Use

Nearly all MS patients possessed a mobile phone (553/576, 96.0%) but older patients used it less frequently (*P *< .001) and less extensively (calling: *P *= .002; text messaging: *P *< .001; browsing websites: *P *< .001; reading or sending emails: *P *= .001; and scheduling: *P *< .001) (Table 3). Women showed a greater tendency for text messaging (*P *< .001) but men were more likely to operate smartphone abilities such as browsing websites via mobile Internet (*P *= .01) or scheduling appointments (*P *= .03).

### Acceptance of Electronic Communication With Health Care Providers

When asked about the acceptability of using various modes of communication, including electronic communication devices, for receiving information and guidance from their physician for managing their MS, 20.5% (120/586) of MS patients accepted communication by mobile phone Internet application or short message service, 41.0% (240/586) by website, 54.3% (318/586) by email, and 67.8% (397/586) by at least one of these modes of communication (Table 4). The majority of patients (539/586, 92.0%) found existing traditional methods acceptable. Acceptance of email services for this purpose exceeded the acceptance of telephone conversations (263/586, 44.9%; *P *= .007). Women were more likely to accept communication by telephone (*P *= .01).

We included media-specific items and demographic variables in all cross-sectional logistic regression models resulting in admissible model fits (Hosmer-Lemeshow test for email: χ^2^_8 _= 7.0, *P *= .54; for websites: χ^2^_8 _= 6.7, *P *= . 58; and for mobile phone features: χ^2^_8 _= 9.2, *P *= .32). Neither sex nor age had an impact on the acceptance of any type of electronic communications media. We eliminated duration of disease from the analyses to avoid multicollinearity, since it was highly correlated with age.

In general, patients with regular a priori use of new electronic media were more likely to accept this form of communication with health care providers (Table 5). More specifically, reading emails at least once a week, browsing Internet websites at least several times a week, and using a mobile phone daily raised the level of acceptance significantly.

Furthermore, online health information seekers (odds ratio 2.4) and patients having already communicated with their physician through a website (odds ratio 3.3) showed a greater interest in website-based communication (Table 5). Scheduling appointments on a mobile phone (odds ratio 2.1) was the only specific task that raised the likelihood of accepting mobile phone features for communication with health care providers.

**Table 3:** Mobile phone use by patients with multiple sclerosis (n = 586)^a^.

Mobile phone use		%	n
**Mobile phone ownership**		96.0	553	
	Missing answers (item 15)		10
**Frequency of mobile phone use**			
	Several times a day	38.0	219
	Once a day	17.5	101
	Several times a week	21.5	124
	Once a week	7.6	44
	Rarely or never	14.2	82
	Missing answers (item 16)		16
**Type of regular mobile phone use**			
	Calling	89.1	513
	Text messaging	63.9	368
	Scheduling	21.7	125
	Audio or video messaging	5.2	30
	Reading or sending emails	4.9	28
	Browsing websites	4.7	27
	Missing answers (item 17)		16

^a ^For all data percentages were based on valid answers.

**Table 4:** Acceptance of modes of communication with health care providers for being informed and instructed during multiple sclerosis therapy (MS) by patients with MS (n = 586).

Type of communication	%	n
By physician (in-person)	92.0	539
By email or mobile phone or website	67.8	397
By email	54.3	318
By telephone call	44.9	263
By website	41.0	240
Via mobile Internet application or short message service	20.5	120

**Table 5:** Acceptance of new media types for communication with health care providers by patients with multiple sclerosis (MS).

Characteristics		Odds ratio	95% confidence interval	*P *value^a^
**Acceptance of emails (n = 581)**				
	Reading emails several times a day	16.3	4.8–55.1	<.001
	Reading emails daily	13.1	4.2–41.0	<.001
	Reading emails several times a week	13.3	4.2–41.8	<.001
	Reading emails once a week	7.0	2.4–20.3	<.001
	Reading emails rarely or never	1 (reference)		
	Sending emails several times a day	1.0	0.4–2.6	.97
	Sending emails daily	1.4	0.6–3.7	.45
	Sending emails several times a week	1.4	0.6–3.3	.43
	Sending emails once a week	0.8	0.4–1.8	.53
	Sending emails rarely or never	1 (reference)		
	Age	1.0	0.8–1.2	.88
	Females	1.0	0.7–1.5	.95
	Males	1 (reference)		
**Acceptance of Internet websites (n = 581)**				
	Browsing websites several times a day	5.3	1.8–15.8	.003
	Browsing websites daily	5.2	1.8–15.4	.003
	Browsing websites several times a week	5.7	1.9–16.8	.002
	Browsing websites once a week	3.1	0.9–10.7	.07
	Browsing websites rarely or never	1 (reference)		
	Using the Internet for browsing	1.3	0.7–2.3	.45
	Using the Internet for chatting	1.4	0.8–2.7	.30
	Using the Internet for video chatting	1.5	0.9–2.7	.13
	Using the Internet for information about MS	2.4	1.7–3.5	<.001
	Using the Internet for communication with physicians	3.3	1.1–10.1	.03
	Using the Internet for communication with other MS patients	1.9	0.8–4.5	.15
	Age	1.1	0.9–1.3	.49
	Females	1.2	0.8–1.8	.39
	Males	1 (reference)		
**Acceptance of mobile Internet applications or short message service (n = 565)**				
	Using a mobile phone several times a day	9.3	2.0–41.7	.004
	Using a mobile phone daily	5.9	1.3–27.3	.02
	Using a mobile phone several times a week	2.9	0.6–13.7	.17
	Using a mobile phone once a week	1.3	0.2–9.6	.81
	Using a mobile phone rarely or never	1 (reference)		
	Using a mobile phone for calling	6.9	0.9–52.4	.06
	Using a mobile phone for text messaging	1.3	0.8–2.3	.32
	Using a mobile phone for audio or video messaging	1.4	0.6–3.2	.45
	Using a mobile phone for browsing websites	1.3	0.4–4.2	.62
	Using a mobile phone for sending and reading emails	0.5	0.2–1.5	.22
	Using a mobile phone for scheduling	2.1	1.3–3.6	.002
	Age	0.9	0.7–1.2	.59
	Females	1.2	0.8–2.0	.37
	Males	1 (reference)		

^a^ *P *< .05 was considered significant.

## Discussion

In this survey, we analyzed the a priori use of computers, the Internet, emails, and mobile phones by patients with MS, as well as their willingness to adopt them for communication with health care providers. Our results indicate that the use of new communication technologies (computers, websites, emails, and mobile phones) by MS patients is already widespread. In addition, the majority of patients reported relevant information and communication technology skills such as installing software. Sharing information and receiving guidance in MS management via email and via website was well accepted among MS patients, with lower levels of acceptability for communication via mobile phone features (mobile Internet applications or short message service). A priori use was the most important predictor of accepting new media for communication with health care providers. Although differences in the a priori use of electronic communication devices between men and women and between younger and older patients were significant, neither sex nor age had an impact on the acceptance of these tools for communicating with health care providers.

### Communication Technology Use by MS Patients

The vast majority of MS patients had access to a computer or owned one themselves. They were regularly visiting websites, and reading and writing emails, and possessed a mobile phone, consistent with results of smaller MS-focused studies [26,31]. The number of Internet users was slightly higher than in more general reports from the Pew Internet & American Life Project [4] but corresponded to the number from Lejbkowicz et al [31]. In agreement with Green et al [38], our study found less-intensive and less-diversified use of computers and Internet services among older people. Our results indicated that women used computers, the Internet, and email services less often, but the prevalence of female nonusers was similar to that of male nonusers. Similarly, Kummervold et al [6] reported an increasing use of the Internet by females in Europe between 2005 and 2007. Mobile phones were the most commonly used devices. As anticipated, young male patients were more likely to use smartphone abilities such as mobile Internet or appointment scheduling.

In our study, 4 in 10 MS patients were already using the Internet regularly for health information seeking and other disease-related tasks. In comparison, 56% to 61% of Americans searched online for health information [3,4]. However, differences between study findings in the use of eHealth services by chronically ill patients may be due to how the studies define media use as described by Wagner et al [39], indicating that 11% accessed health information services at least monthly but 45.9% had ever used them in the previous year [2]. Several European studies noted an increasing rate of Internet use for health purposes, with the highest increase being in Germany (from 44.4% in 2005 to 56.6% in 2007) [6,8,9]. In contrast to Santana et al [8], we did not find any differences in the use of new media with respect to place of residence.

In contrast to Hay et al [11], who reported that 82% of MS patients retrieved medical information online before the first visit, our rates were lower. Lejbkowicz et al [31] observed that 63% of MS patients used the Internet for MS-related tasks, but they did not state how they obtained that information. Nonetheless, only a minority of MS patients used the opportunity to correspond with their physician or other patients via the Internet. Although other studies reported similar results, evidence for increasing interest in online health care services is growing [2,6,8,9,40].

### Acceptance of Electronic Communication with Health Care Providers

In addition to the a priori use of electronic communication devices, we investigated the a priori attitude toward adopting these services for aiding in MS disease management. Most participants accepted more than one type of new media for communication with health care providers. Communication via email and website was considered as acceptable as conventional telephone calls. A notable proportion of patients perceived the integration of mobile phones and mobile Internet to aid their therapy as useful. Two-thirds of MS patients were willing to use at least one new media service for communication with health care providers. The main factor in accepting a service was a priori use. Patients who read emails at least once a week, browsed websites several times a week, and used a mobile phone daily were more likely to approve a type of communication. Likewise, current users of electronic communication devices for health care management showed a greater interest in Web-based communication.

Several studies support the open-mindedness of patients toward using email communication in disease management. Hassol et al [16] reported that patients who used electronic health records generally preferred emails to telephone calls. In a study focusing on older patients, Singh et al [40] found that 49.3% of them could imagine using emails for communication with their physician. In agreement with our findings, a priori use of email services was the best predictor for acceptance of email communication with health care providers [40]. Santana et al [9] reported that about 18% of Europeans usually contact their physician via Internet and 25% would like to make their appointments online. Moreover, 4 out of 10 people in Europe would select their physician with regard to eHealth services provided [9]. Similarly, 49% of Israeli MS patients welcomed the opportunity to communicate with their medical team online [31].

### Limitations

Some limitations of our survey warrant consideration. Participation in the survey was nonrestricted and hence may have introduced selection bias by motivation or interest. However, this was minimized by the large number of participants. Moreover, relevant demographic data, such as income or ethnicity, that other studies had used were lacking in our survey. Therefore, we were unable to test assumptions about potential obstacles posed by new media such as the digital divide. As income is a sensitive issue, participants may not have been willing to specify their personal particulars. We conducted our survey using paper-and-pencil questionnaires to avoid selection bias. Differences in the methods of similar studies in defining and assessing media use are important when attempting to compare studies on the use of health care information and communication technology. The questionnaire provided only a single item for the acceptability of new communication methods with health care providers, which may have been insufficient to obtain nuance and detail about the patients’ true attitudes. We surveyed attitudes toward electronic communication devices but lacked data on actual behavior or objective use.

### Conclusions

In our survey, we obtained data on the a priori use of new media by chronically ill patients with MS, as well as their attitudes toward future use of new media for communication with health care providers and differentiating between common communication types.

To summarize, our results indicate that the majority of MS patients are willing to use new media for further eHealth implementations. Although the potential benefits (and risks) of using electronic communication devices in MS health care services remain to be established, our data suggest these tools can be integrated with electronic health records and patient relationship management systems in order to increase the range of potential users and capabilities.

We agree with Nijland et al [41] that delivering high-quality health care to patients in as many suitable ways as possible should make a significant impact on the design of eHealth applications. eHealth services such as patient relationship management systems and electronic health records that focus on only a single communication type will miss the chance to maximize their effectiveness for use in the health care process. According to our findings, there are two gaps. First, although 40% to 50% of patients would like to use eHealth services to aid their therapy, only 5% of patients currently use them. Second, 90% of patients are technically skilled enough to use information and communication technology but only 50% are willing to use health information and communication technology. To overcome these obstacles, the design and implementation of eHealth applications in the health care process have to be tailored to patients’ individual needs. Further research should focus on educational content as well as on technical options to deliver the content to those who would benefit from it.

## Multimedia Appendix 1

English version of the new media questionnaire.

## Multimedia Appendix 2

Results of the survey on the use and acceptance of electronic communication by patients with multiple sclerosis.

## Multimedia Appendix 3

English version of the new media questionnaire.

## Abbreviations

MS: multiple sclerosis

## References

[1] Eysenbach G, Kohler Ch (2003). What is the prevalence of health-related searches on the World Wide Web? Qualitative and quantitative analysis of search engine queries on the internet. AMIA Annu Symp Proc.

[2] Baker L, Wagner TH, Singer S, Bundorf MK (2003). Use of the Internet and e-mail for health care information: results from a national survey. JAMA.

[3] Ybarra ML, Suman M (2006). Help seeking behavior and the Internet: a national survey. Int J Med Inform.

[4] Fox S, Jones S (2009). The social life of health information.

[5] (2003). Gesundheitsberichterstattung des Bundes.

[6] Kummervold PE, Chronaki CE, Lausen B, Prokosch HU, Rasmussen J, Santana S, Staniszewski A, Wangberg SC (2008). eHealth trends in Europe 2005-2007: a population-based survey. J Med Internet Res.

[7] Andreassen HK, Bujnowska-Fedak MM, Chronaki CE, Dumitru RC, Pudule I, Santana S, Voss H, Wynn R (2007). European citizens' use of E-health services: a study of seven countries. BMC Public Health.

[8] Santana S, Lausen B, Bujnowska-Fedak M, Chronaki CE, Prokosch HU, Wynn R (2011). Informed citizen and empowered citizen in health: results from an European survey. BMC Fam Pract.

[9] Santana S, Lausen B, Bujnowska-Fedak M, Chronaki C, Kummervold PE, Rasmussen J, Sorensen T (2010). Online communication between doctors and patients in Europe: status and perspectives. J Med Internet Res.

[10] Kobelt G, Berg J, Lindgren P, Elias WG, Flachenecker P, Freidel M, König N, Limmroth V, Straube E (2006). Costs and quality of life of multiple sclerosis in Germany. Eur J Health Econ.

[11] Hay MC, Strathmann C, Lieber E, Wick K, Giesser B (2008). Why patients go online: multiple sclerosis, the internet, and physician-patient communication. Neurologist.

[12] Nielsen AS, Halamka JD, Kinkel RP (2012). Internet portal use in an academic multiple sclerosis center. J Am Med Inform Assoc.

[13] Ziemssen T (2011). Symptom management in patients with multiple sclerosis. J Neurol Sci.

[14] Harland J, Bath P (2007). Assessing the quality of websites providing information on multiple sclerosis: evaluating tools and comparing sites. Health Informatics J.

[15] Hoffmann T, Twork S, Pöhlau D, Kugler J (2009). Patientenorientierung im Internet: qualitative Bewertung von Internetseiten für Multiple-Sklerose-Betroffene. Akt Neurol.

[16] Hassol A, Walker JM, Kidder D, Rokita K, Young D, Pierdon S, Deitz D, Kuck S, Ortiz E (2004). Patient experiences and attitudes about access to a patient electronic health care record and linked web messaging. J Am Med Inform Assoc.

[17] Lowe-Strong A, McCullagh PJ (2005). Monitoring of symptoms and interventions associated with multiple sclerosis. Stud Health Technol Inform.

[18] Michel-Verkerke MB, Schuring RW, Spil TAM (2004). Workflow management for multiple sclerosis patients: IT and organization. Proceedings of the 37th Annual Hawaii International Conference on Systems Sciences.

[19] Schultheiss T, Kempcke R, Kratzsch F, Eulitz M, Pette M, Reichmann H, Ziemssen T (2012). [Multiple sclerosis management system 3D. Moving from documentation towards management of patients]. Nervenarzt.

[20] Hatzakis MJ, Allen C, Haselkorn M, Anderson SM, Nichol P, Lai C, Haselkorn JK (2006). Use of medical informatics for management of multiple sclerosis using a chronic-care model. J Rehabil Res Dev.

[21] Fernandez-Luque L, Elahi N, Grajales FJ (2009). An analysis of personal medical information disclosed in YouTube videos created by patients with multiple sclerosis. Stud Health Technol Inform.

[22] Wicks P, Massagli M, Kulkarni A, Dastani H (2011). Use of an online community to develop patient-reported outcome instruments: the Multiple Sclerosis Treatment Adherence Questionnaire (MS-TAQ). J Med Internet Res.

[23] Goverover Y, O'Brien AR, Moore NB, DeLuca J (2010). Actual reality: a new approach to functional assessment in persons with multiple sclerosis. Arch Phys Med Rehabil.

[24] Negreiros MA, Mattos P, Landeira-Fernandez J, Paes RA, Alvarenga RP (2008). A brief neuropsychological screening test battery for cognitive dysfunction in Brazilian multiple sclerosis patients. Brain Inj.

[25] Younes M, Hill J, Quinless J, Kilduff M, Peng B, Cook SD, Cadavid D (2007). Internet-based cognitive testing in multiple sclerosis. Mult Scler.

[26] Atreja A, Mehta N, Miller D, Moore S, Nichols K, Miller H, Harris CM (2005). One size does not fit all: using qualitative methods to inform the development of an Internet portal for multiple sclerosis patients. AMIA Annu Symp Proc.

[27] Matti AI, McCarl H, Klaer P, Keane MC, Chen CS (2010). Multiple sclerosis: patients' information sources and needs on disease symptoms and management. Patient Prefer Adherence.

[28] Ball MJ, Lillis J (2001). E-health: transforming the physician/patient relationship. Int J Med Inform.

[29] Wald HS, Dube CE, Anthony DC (2007). Untangling the Web--the impact of Internet use on health care and the physician-patient relationship. Patient Educ Couns.

[30] Lode K, Larsen JP, Bru E, Klevan G, Myhr KM, Nyland H (2007). Patient information and coping styles in multiple sclerosis. Mult Scler.

[31] Lejbkowicz I, Paperna T, Stein N, Dishon S, Miller A (2010). Internet usage by patients with multiple sclerosis: implications to participatory medicine and personalized healthcare. Mult Scler Int.

[32] Pucci E (2003). Is the internet transforming the physician-consumer relationship? Preliminary data in a neurological setting. Eur J Neurol.

[33] Gerber BS, Eiser AR (2001). The patient physician relationship in the Internet age: future prospects and the research agenda. J Med Internet Res.

[34] Polman CH, Reingold SC, Edan G, Filippi M, Hartung HP, Kappos L, Lublin FD, Metz LM, McFarland HF, O'Connor PW, Sandberg-Wollheim M, Thompson AJ, Weinshenker BG, Wolinsky JS (2005). Diagnostic criteria for multiple sclerosis: 2005 revisions to the "McDonald Criteria". Ann Neurol.

[35] Statistisches Landesamt Baden-Wuerttemberg, Germany (2011). Statistische Berichte Baden-Wuerttemberg.

[36] Statistisches Landesamt des Freistaates Sachsen, Germany (2012). www.statistik.sachsen.de.

[37] Minden SL, Frankel D, Hadden L, Hoaglin DC (2007). Access to health care for people with multiple sclerosis. Mult Scler.

[38] Green BB, Anderson ML, Ralston JD, Catz S, Fishman PA, Cook AJ (2011). Patient ability and willingness to participate in a web-based intervention to improve hypertension control. J Med Internet Res.

[39] Wagner TH, Baker LC, Bundorf MK, Singer S (2004). Use of the Internet for health information by the chronically ill. Prev Chronic Dis.

[40] Singh H, Fox SA, Petersen NJ, Shethia A, Street RL (2009). Older patients' enthusiasm to use electronic mail to communicate with their physicians: cross-sectional survey. J Med Internet Res.

[41] Nijland N, van Gemert-Pijnen J, Boer H, Steehouder MF, Seydel ER (2008). Evaluation of internet-based technology for supporting self-care: problems encountered by patients and caregivers when using self-care applications. J Med Internet Res.

