# Supplementary material for: Use and Acceptance of Electronic Communication by Patients With Multiple Sclerosis: A Multicenter Questionnaire Study
Source: J Med Internet Res. 2012 Oct 15;14(5):e135. doi: 10.2196/jmir.2133 (PMC3510727; doi:10.2196/jmir.2133)
Supplement: Supplementary file 1 [file jmir_v14i5e135_app1.pdf]

Multimedia Appendix 1. English version of the new media questionnaire (18 items).

Number	Question
**Demographics**	
01	How old are you?
02	What is your gender?
	a) male
	b) female
03	What is your postal code?
04	When were you diagnosed with MS (year)?
**Computer use**	
05	How often do you usually use a computer?
	a) Several times a day
	b) Once a day
	c) Several times a week
	d) Once a week
	e) Rarely / never
06	Do you own a computer?
	a) Yes
	b) Yes – I've got a shared computer access
	c) No
07	What do you regularly use your computer for? (Multiple answers possible)
	a) Word processing
	b) Emailing
	c) Browsing websites
	d) Chatting
	e) Getting information about MS
08	Are you able to acquaint yourself quickly with a new computer program?
	a) Definitely applies to me
	b) Mostly applies to me
	c) Slightly applies to me
	d) Does not apply to me
09	Have you already installed computer programs on your own?
	a) Yes
	b) No

Internet use

10 How often do you usually browse websites on the Internet?

a) Several times a day

b) Once a day

c) Several times a week

d) Once a week

e) Rarely / never

11 What type of Internet access do you have at home?

a) Broadband / high speed access

b) Low speed access (e.g. via modem or ISDN)

c) I have no Internet access

d) I have Internet access but I don’t know the type of access

12 What do you regularly use the Internet for? (Multiple answers possible)

a) Browsing websites

b) Chatting

c) Video chatting

d) Getting information about MS

e) Communicating with physicians

f) Communicating with other MS patients

13 How often do you send emails?

a) Several times a day

b) Once a day

c) Several times a week

d) Once a week

e) Rarely / never

14 How often do you read emails?

a) Several times a day

b) Once a day

c) Several times a week

d) Once a week

e) Rarely / never

Mobile phone use

15 Do you own a mobile phone?

a) Yes

b) No

16 How often do usually use a mobile phone?

a) Several times a day

b) Once a day

c) Several times a week

d) Once a week

e) Rarely / never

17 What do you regularly use a mobile phone for? (Multiple answers possible)

a) Calling

b) Text messaging

c) Audio / video messaging

d) Browsing websites

e) Reading / sending emails

f) Scheduling

Acceptance

18 What type of communication would you accept for being informed and instructed during your MS therapy?

a) By physician

b) By telephone call

c) Via mobile Internet application or short message service

d) By website

e) By email
