# Supplementary material for: Use and Acceptance of Electronic Communication by Patients With Multiple Sclerosis: A Multicenter Questionnaire Study
Source: J Med Internet Res. 2012 Oct 15;14(5):e135. doi: 10.2196/jmir.2133 (PMC3510727; doi:10.2196/jmir.2133)
Supplement: Supplementary file 2 [file jmir_v14i5e135_app2.pdf]

Multimedia Appendix 2. Results of the survey on the use and acceptance of electronic communication by patients with multiple sclerosis. For all data percentages were based upon valid answers. Five patients did not disclose their age.

	All patients (n= 586)		Male (n = 178)		Female (n = 408)		Younger patients[a] (n = 283)		Older patients[b] (n = 298)	
	%	n	%	n	%	n	%	n	%	n
Using a computer										
Several times a day	46.6	273	60.7	108	40.4	165	53.4	151	40.6	121
Daily	24.2	142	18.0	32	27.0	110	24.4	69	24.2	72
Several times a week	15.0	88	12.4	22	16.2	66	14.5	41	15.4	46
Once a week	5.3	31	1.7	3	6.9	28	3.9	11	6.4	19
Rarely / never	8.9	52	7.3	13	9.6	39	3.9	11	13.4	40
Owning a computer OR having a shared computer access	95.2	558	94.9	169	95.3	398	95.8	271	94.6	282
Owning a computer	75.4	442	84.8	151	71.3	291	79.9	226	71.5	213
Using a computer regularly for										
Word processing	62.6	367	70.2	125	59.3	242	65.4	185	61.1	182
Emailing	81.7	479	82.0	146	81.6	333	86.9	246	77.5	231
Browsing the Internet	81.9	480	80.3	143	82.6	337	86.2	244	77.5	231
Chatting	12.5	73	16.9	30	10.5	43	17.7	50	7.7	23
Getting information about MS	39.6	232	34.8	62	41.7	170	40.6	115	38.9	116
Acquainting quickly with a new computer program										
Definitely applies to me	29.0	170	36.0	64	26.0	106	35.3	100	23.2	69
Mostly applies to me	33.6	197	32.6	58	34.1	139	37.5	106	30.2	90
Slightly applies to me	24.2	142	19.1	34	26.5	108	19.4	55	28.5	84
Does not apply to me	13.1	77	12.4	22	13.5	55	7.8	22	18.5	55
Having already installed computer programs	64.4	375	80.9	144	56.6	231	73.9	209	54.7	163
Browsing websites on the Internet										
Several times a day	34.6	203	44.9	80	30.1	123	42.8	121	27.5	82
Once a day	25.9	152	24.2	43	26.7	109	24.4	69	27.2	81
Several times a week	23.2	136	18.0	32	25.5	104	22.6	64	23.8	71
Once a week	6.1	36	3.9	7	7.1	29	4.6	13	7.4	22
Rarely / never	10.1	59	9.0	16	10.5	43	5.7	16	14.1	42
Having an Internet access at home										
Broadband access	69.6	408	78.1	139	65.9	269	74.2	210	65.1	194
Low speed access	20.1	118	14.0	25	22.8	93	15.9	45	24.2	72
No access	6.0	35	6.2	11	5.9	24	5.7	16	6.4	19
Access type unknown	4.3	25	1.7	3	5.4	22	4.2	12	4.4	13
Using the Internet regularly for										
Browsing websites	81.4	477	81.5	145	81.4	332	86.2	244	77.2	230
Chatting	10.2	60	15.7	28	7.8	32	15.2	43	5.7	17
Video chatting	11.1	65	14.6	26	9.6	39	14.8	42	7.7	23
Getting information about MS	37.2	218	36.0	64	37.7	154	38.9	110	35.2	105
Communicating with physician	3.8	22	3.9	7	3.7	15	4.9	14	2.7	8
Communicating with other MS patients	4.9	29	6.2	11	4.4	18	6.7	19	3.4	10

Sending emails										
Several times a day	31.1	182	39.3	70	27.5	112	35.0	99	27.9	83
Once a day	15.4	90	16.3	29	15.0	61	16.6	47	14.4	43
Several times a week	23.0	135	16.9	30	25.7	105	21.9	62	23.8	71
Once a week	11.6	68	10.1	18	12.3	50	13.8	39	9.7	29
Rarely / never	18.9	111	17.4	31	19.6	80	12.7	36	24.2	72
Reading emails										
Several times a day	37.2	218	47.2	84	32.8	134	42.8	121	32.6	97
Once a day	23.2	136	21.3	38	24.0	98	22.3	63	23.8	71
Several times a week	17.4	102	9.6	17	20.8	85	16.3	46	18.5	55
Once a week	9.2	54	10.1	18	8.8	36	9.9	28	8.4	25
Rarely / never	13.0	76	11.8	21	13.5	55	8.8	25	16.8	50
Owning a mobile phone	96.0	553	96.0	169	96.0	384	97.8	271	94.2	277
Missing answers (item 15)		10		2		8		6		4
Using a mobile phone										
Several times a day	38.0	219	43.2	76	36.3	143	54.0	148	23.7	69
Once a day	17.5	101	16.5	29	18.3	72	16.4	45	18.9	55
Several times a week	21.5	124	20.5	36	22.3	88	20.1	55	23.4	68
Once a week	7.6	44	6.8	12	8.1	32	3.6	10	11.7	34
Rarely / never	14.2	82	13.1	23	15.0	59	5.8	16	22.3	65
Missing answers (item 16)		16		2		14		9		7
Using a mobile phone regularly for										
Calling	89.1	513	91.5	161	88.0	352	93.1	258	85.0	250
Text messaging	63.9	368	52.3	92	69.0	276	78.0	216	50.7	149
Audio / video messaging	5.2	30	5.1	9	5.3	21	6.9	19	3.7	11
Browsing websites	4.7	27	8.0	14	3.3	13	7.9	22	1.7	5
Reading / sending emails	4.9	28	7.4	13	3.8	15	7.9	22	2.0	6
Scheduling	21.7	125	27.3	48	19.3	77	29.2	81	14.3	42
Missing answers (item 17)		16		2		14		9		7
What type of communication would you accept for being informed and instructed during your MS therapy?										
By physician	92.0	539	91.6	163	92.2	376	92.2	261	91.6	273
By telephone call	44.9	263	37.1	66	48.3	197	47.3	134	42.6	127
Via mobile Internet application or short message service	20.5	120	24.2	43	18.9	77	27.2	77	13.8	41
By website	41.0	240	39.3	70	41.7	170	44.2	125	37.6	112
By email	54.3	318	54.5	97	54.2	221	57.2	162	51.7	316
By email or mobile phone or website	67.7	397	70.8	126	66.4	271	72.4	205	63.4	189

[a] Patients between the ages of 17 and 40

[b] Patients between the ages of 41 and 73
